# Deliberate self-harm behavior among young violent offenders

**DOI:** 10.1371/journal.pone.0182258

**Published:** 2017-08-17

**Authors:** Natalie Laporte, Andrejs Ozolins, Sofie Westling, Åsa Westrin, Eva Billstedt, Björn Hofvander, Märta Wallinius

**Affiliations:** 1 Lund University, Faculty of Medicine, Department of Clinical Sciences Lund, Unit for Clinical Suicide Research, Lund, Sweden; 2 Linneaus University, Department of Psychology, Växjö, Sweden; 3 Sahlgrenska Academy, Institute of Neuroscience and Physiology, Gillberg Neuropsychiatry Centre, Gothenburg, Sweden; 4 Lund University, Faculty of Medicine, Department of Clinical Sciences Lund, Child and Adolescent Psychiatry, Lund, Sweden; University of Toronto, CANADA

## Abstract

Deliberate self-harm behavior (DSH) can have profound effects on a person’s quality of life, and challenges the health care system. Even though DSH has been associated with aggressive interpersonal behaviors, the knowledge on DSH in persons exhibiting such behaviors is scarce. This study aims to (1) specify the prevalence and character of DSH, (2) identify clinical, neurocognitive, psychosocial, and criminological characteristics associated with DSH, and (3) determine predictors of DSH among young violent offenders. Data were collected from a nationally representative cohort of 270 male violent offenders, 18–25 years old, imprisoned in Sweden. Participants were interviewed and investigated neuropsychologically, and their files were reviewed for psychosocial background, criminal history, mental disorders, lifetime aggressive antisocial behaviors, and DSH. A total of 62 offenders (23%) had engaged in DSH at some point during their lifetime, many on repeated occasions, yet without suicidal intent. DSH was significantly associated with attention deficit hyperactivity disorder, mood disorders, anxiety disorders, various substance use disorders, being bullied at school, and repeated exposure to violence at home during childhood. Mood disorders, anxiety disorders, and being bullied at school remained significant predictors of DSH in a total regression model. Violent offenders direct aggressive behaviors not only toward other people, but also toward themselves. Thus, DSH must be assessed and prevented in correctional institutions as early as possible, and more knowledge is needed of the function of DSH among offenders.

## Introduction

Self-harm is a serious self-destructive behavior that implies psychological suffering, can have long-term and profound effects on a person’s quality of life, and challenges the health care system [1, 2]. It is also demanding on close friends and family. The risk of completed suicide among people who self-harm is 30 times higher than among those who do not [3].

### Definitions

Defining self-harm is a challenge, and a variety of definitions have been proposed. We chose to define deliberate self-harm (DSH) according to Hawton & James as ranging from “behaviors with no suicidal intent (but with the intent to communicate distress or relieve tension) through to suicide” [4] (p. 891). Acts typical of DSH include cutting, burning, biting, scratching or excessively rubbing the skin, self-hitting, head-banging or hitting fists against objects, ingesting a substance, drug, or object and jumping from a height with the intent of causing self-harm [5]. They also include bone-breaking, interfering with wound healing, and hair pulling [6].

### Prevalence

In the general population, the international prevalence of lifetime DSH is estimated at 4% to 13% [7, 8]. Deliberate self-harm is prevalent in all ages, but peaks in adolescence (15–17 years) [9]. In a study sample of 1088 adolescents, DSH was prevalent among 36% [10]. In another study of 1052 adolescents (13–14 years), 42% reported DSH [11]. Previous studies on adolescents have found gender differences in DSH, with a higher prevalence in girls than boys [12]. However, several studies have not found gender differences, and the current view is that self-harm is more common among men than previously thought [13]. In 2012, 804 000 individuals committed suicide worldwide [14]. Suicide rates in prison have been reported to be three to six times higher than in the general population [15, 16]. In one study the risk of suicide was higher in those who self-harmed than in the general prison population, and more than half of the deaths occurred within a month of self-harming [17]. Other studies report that DSH precedes suicide in prison environments in 43% to 50% of cases [18, 19].

### Risk factors

DSH has been associated with general psychopathology [11], and mental disorders have been described as the strongest predictors of suicide attempts [20]. High rates of anxiety, depression, impulse control disorders, and mental retardation have been observed among individuals with DSH in a general population [21, 22]. Substance use disorders (SUD) have also been related to a higher number of self-harm incidents [23]. Several studies have also found a link between attention deficit hyperactivity disorder (ADHD) and DSH [24]. Psychosocial background is also associated with DSH. Studies have found that childhood abuse increases the risk of DSH in adolescence and adulthood [25, 26,27], and recent bullying victimization and expulsion from school have also been associated with DSH [28].

### Aims

DSH is a challenging, self-destructive behavior that has been associated with a variety of mental disorders and early adverse experiences. However, previous research on DSH has mostly targeted a selected population of young, Caucasian women or children and adolescents [29]. As DSH can be viewed as self-inflicted aggressive behavior [30], a (biological) connection to interpersonal aggressive behavior has been suggested and reported [31, 32]. Yet, knowledge about DSH in persons exhibiting interpersonal aggressive behaviors is scarce, especially among offenders past adolescence.

The aims of this study are to

- specify the prevalence and character of DSH among young adult violent male offenders;
- identify clinical, neurocognitive, psychosocial, and criminological characteristics associated with DSH among young adult violent male offenders; and
- determine predictors of DSH among young adult violent male offenders.

## Methods

### Participants

The study cohort were young adult (18–25 years of age) male offenders incarcerated between March 2010 and July 2012 at any of nine correctional facilities in the Western region of the Swedish Prison and Probation Service for committing violent (including “hands-on” sexual) offenses. The region has the full range of prisons, from high-security to open facilities, and serves approximately one fifth of the total national cohort. Because there is only one small, specialized women’s prison in the defined area, female offenders were not included in the study. Exclusion criteria were: poor fluency in Swedish, defined as the need for an interpreter for full participation, and duration of stay in the prison of 4 weeks or less. To assess the representativeness of the included group, demographic information was collected for individuals who were excluded or chose not to participate in the study.

Of a total of 421 offenders, 23 (5%) were excluded because of insufficient language skills and 19 (5%) for insufficient duration of prison stay. Of the remaining 379 offenders, 109 (29%) declined participation in the study, leaving a final study group of 270 participants (71% of all who met inclusion criteria). Age ranged from 18 years and 7 months to 25 years and 11 months, with a mean age of 22.3 years (*SD* = 1.9). Fifteen (14%) had been sentenced for sexual violent crimes, and 94 (86%) for non-sexual violent crimes. Those excluded for insufficient skills in Swedish differed from the participants by having a higher rate of sexual crimes (n = 12; 52%). The cohort is considered representative of young adult male violent offenders in the Swedish Prison and Probation Service.

Detailed information on the participants’ psychosocial background has been reported [33]. Of the 270 participants, 196 (73%) were born in Sweden, but 114 (43%) reported that both parents had been born outside of Sweden. At some point in their childhood or adolescence 107 participants (40%) had been placed in an institution, and the majority (n = 231; 86%) had been convicted of crimes previously. Mental disorders, including childhood onset mental disorders and SUD, were common but only a few participants had undergone assessment at a child and adolescent psychiatry unit [34, 35].

### Procedures

After receiving oral and written information on the study, eligible offenders were asked for informed consent. A small monetary compensation for time spent in the study was provided (SEK 200, approximately 21€). Participants were assessed consecutively according to a preset protocol that included self-rated questionnaires, semi-structured diagnostic interviews, and neuropsychological assessments. Questionnaires were completed by the participants prior to the clinical assessments, which were subsequently performed over a full day by a licensed psychologist with clinical experience in the field and special training in the instruments used. The assessor had read all the file information on each participant, including prison health care journals, detailed reports on previous living circumstances and criminal history, and incidents during current incarceration that was available from the Swedish Prison and Probation Service. All clinical data were assessed for quality and diagnoses were assigned by consensus between the assessor and one senior clinician and researcher with considerable experience in the field. Participants who showed signs of an autism spectrum disorder were later offered a specialized clinical, semi-structured diagnostic interview or observational assessment.

### Measures

#### DSH

Information on lifetime DSH was collected from files and interviews, separately for non-suicidal self-injuries (any occasion, nr. of occasions, age at onset, type of self-injury) and suicide attempts (any attempt, nr. of attempts, age at onset, violent attempts, intention of most serious attempt, risk of completed suicide at most serious attempt). A merged variable, DSH, was then created based on the occurrence of either non-suicidal self-injury (NSSI) or suicide attempts.

#### Mental disorders

The lifetime occurrence of categorical diagnoses and dimensional symptoms of mental disorders was assessed according to the Diagnostic and Statistical Manual of Mental Disorders, 4th edition (DSM IV) [36], based on information from the Structured Clinical Interview guides for Axis I and II disorders (SCID I and SCID II) [37, 38] and file information provided by the Swedish Prison and Probation Service. Symptoms of autism spectrum disorders and other neurodevelopmental disorders (e.g., ADHD) were measured using the Asperger syndrome/high functioning autism diagnostic interview [39] and a structured DSM-IV interview protocol that has been used in similar studies [40].

#### Cognitive functioning

The General Ability Index (GAI) [41] from the Wechsler Adult Intelligence Scale–third edition (WAIS-III) [42] was used to assess participants’ general intelligence. The GAI consists of two domains: the verbal comprehension index (VCI) measures acquired knowledge and verbal reasoning, and the perceptual organization index (POI) measures visual-motor skills, nonverbal fluid reasoning, and visuospatial organization.

#### Psychosocial background

Information on ethnicity, education, disadvantaged childhood circumstances, institutionalization during childhood, and previous contacts with the mental health care system was obtained from interviews and file information.

#### Criminal history

The structured research protocol was also used to collect data on criminal history. All earlier criminality (including the index offense) was assembled: offenses noted in the files as well as those confessed during the interviews. If the participant reported a younger age of onset and more criminality than noted in the files, the information from the interviews was used if the assessor considered the information credible. Thus, it was possible to include information on onset of criminality before the age of 15, even though this is not officially registered in Sweden.

Lifetime aggressive antisocial behaviors was also investigated through the Life History of Aggression questionnaire (LHA) [43], originally developed for research on neurobiological correlates to aggression. The LHA evaluates the frequency of 11 different types of aggressive and antisocial behaviors, rated on a 5-point scale. The LHA total score equals the sum of the following subscales: Aggression, Self-directed aggression, and Antisocial behavior [44]. The LHA was administered as a clinician-rated instrument, and the assessor based the ratings on all available information from interviews and files.

### Statistical methods

Data was anonymized and coded, then analyzed with IBM SPSS Statistics 22 software. Screening for outliers was performed. When data were skewed, nonparametric statistics were used, Mann-Whitney U test for all analyses. For all continuous variables, Cohen’s d was calculated and will be presented under each section. A DSH variable was created by merging the groups of self-harming participants and suicide attempters. Due to missing data for some variables, the percentages given in the results section are based on the valid percentages in the analyses. Frequencies presented in Table 1 refers to the total frequencies of mental disorders in the group, therefore the count of DSH yes/no in Table 1 may differ from the total frequencies of mental disorders in the same table due to missing data for some variables. Data for Aim 1 were processed using frequencies and descriptive tables; results are given for the general DSH variable as a whole, and for non-suicidal self-injuries and suicide attempts as separate parts. Aim 2 was analyzed using Fischer’s Exact test, Mann-Whitney U test, and Student’s *t*-test. Some clinical variables, e.g., borderline personality disorder (n = 13), eating disorders (n = 3), dissociative syndromes (n = 2), and intermittent explosivity (n = 10) could not be analyzed within the second aim because of low prevalence in the sample. Aim 3 was investigated using binary logistic regression in two models, simple and multiple, and the multiple model included only potential risk factors/important confounders described in previous studies. As a final step, a multiple model controlling for age was performed. The predictors were screened for multicollinearity with acceptable variance inflation factor values and tolerance. Sensitivity, specificity, and positive predictive value (accuracy) was calculated for childhood ADHD and early life adversities (continuous measure). All bivariate analyses were performed with the general DSH variable as dependent variable.

**Table 1:** Associations between mental disorders and deliberate self-harm behavior

**Mental Disorder**	**Total prevalence (N)**	**Deliberate Self-Harm Behavior (N)**			**Fischer’s Exact *p***
		No	Yes	Expected Yes-Count	
**Mood disorders**	145	90	55	34	.000
**Anxiety disorders**	138	87	50	32	.000
**Psychotic syndromes**	22	13	8	5	.110
**ADHD in childhood, any**	170	122	46	40	.069
**ADHD in adulthood, any**	116	79	37	27	.005
**Autism spectrum disorders**	26	18	7	6	.620
**Conduct disorder**	210	159	49	49	1.0
**Antisocial personality disorder**	170	132	36	39	.367
**Paranoid personality disorder**	26	19	7	6	.631
**Impulse control disorders**	55	39	15	13	.472
**SUD, any**	228	168	55	52	.323
**-Alcohol SUD**	130	89	40	30	.006
**-Sedatives SUD**	130	92	37	30	.042
**-Amphetamine SUD**	130	89	39	30	.008
**-Hallucinogen SUD**	90	61	28	21	.030
**-Cannabis SUD**	208	155	48	48	1.0
**-Cocaine SUD**	106	82	24	25	.883
**-Metadon**	38	23	15	9	.021
**Extremely destructive abuse (drugs/alcohol)**	58	34	24	14	.001
**Borderline intellectual functioning**	53	35	17	12	.100

Note: all *p*-values from Fischer’s Exact test. All diagnoses consider lifetime occurrence, if not otherwise specified.

### Ethical considerations

All offenders provided informed, written consent before participation, and were given the opportunity to receive feedback on the preliminary results from the assessments. Offenders showing indications of severe psychopathology were then given the opportunity to be referred to the prison psychiatrist for continued assessment and treatment. The study, including the monetary reward (which was low in order not to give an incentive that would compromise the free consent), was approved by the Research Ethics Committee at Lund University.

## Results

### Prevalence and character of DSH among young violent offenders

Among the 270 young offenders, a total of 62 (23%) had engaged in DSH at some point. Specifically, 17 offenders stated repetitive self-harming acts (five or more occasions) while 20 offenders had harmed themselves on fewer than five occasions. Four participants reported having harmed themselves, but did not report the number of occasions. Forty-eight offenders (18%) reported suicide attempts: 22 had made one suicide attempt, 25 had made multiple (2–6) attempts, and one participant reported as much as 15 suicide attempts. Among the 48 suicide attempters, only 6 offenders expected the consequence of the attempt to be death. Just as many (n = 6) reported performing the suicide attempt with no or little intention to die, or as a manipulative behavior. The majority of suicide attempters (n = 35) reported an ambivalent intention. Mean age at onset of self-harming was 16 (*Mdn* = 16, *SD* = 3.4; range 5–22) and at onset of suicidal behavior was 17 (*Mdn* = 17, *SD* = 3.6; range 9–25) years.

### Characteristics associated with DSH among young violent offenders

#### Clinical

Table 1 displays associations (Fischer’s Exact test) between mental disorders and DSH, showing that the majority of the DSH participants were diagnosed with mood disorders (89%) and/or anxiety disorders (82%) at some point during their lifetime. Also, differing types of SUD and adult ADHD were associated with DSH. When attention deficit (AD) and hyperactivity disorder (HD) symptoms were analyzed separately as continuous variables using Mann-Whitney U test, DSH participants had significantly more childhood AD symptoms (*p* = .022, Cohen’s *d = 0*.*29*), adult AD symptoms (*p* = .016, Cohen’s *d* = 0.30), and adult HD symptoms (*p* = .02, Cohen’s *d* = 0.28) than those who did not self-harm. There was no significant difference in childhood HD symptoms between DSH participants (*M* = 5.5, *SD* = 2.9) and non-DSH participants (*M =* 4.8, *SD* = 2.8), *p* = .104 (Cohen’s *d* = 0.21).

#### Cognitive functioning

Cognitive abilities were compared between DSH participants and non-DSH participants, and no significant differences were found in GAI, *t*(1,260) = 1.91, *p* = .06, or VCI, *t*(1,260) = 1.02, *p* = .31. However, DSH participants had significantly lower scores on the POI scale than the non-DSH participants, *t*(1,260) = 2.50, *p* = .01 (Cohen’s *d* = 0.31).

#### Psychosocial

Significantly more DSH than non-DSH participants reported having been bullied at school, while no such conclusion could be drawn from participants who bullied others at school (*p* = .000 and *p* = .104, respectively). Having been exposed to violence on repeated occasions during childhood was also significantly related to DSH (*p* = .001). There was no significant association between number of placements at correctional institutions at a young age and DSH (*p* = .280, Cohen’s *d* = .47). When early adverse experiences (exposed to violence on repeated occasions, placed in an institution, parent deceased, parent with SUD or other serious disease) were merged into a dimensional measure of psychosocial adversities during childhood or adolescence, an increased number of such experiences were related to DSH (*p* = .001, Cohen’s *d* = .39).

#### Criminological variables

There were no significant differences between the two groups regarding criminological variables including different types of violent criminality. However, those who had been the subject of forensic psychiatric care reported more DSH (*p* = .003). Assaulting one’s parents during childhood was also associated with DSH (*p* = .013). Participants with DSH had a higher LHA total score (*p* = .001) than those without DSH; however, this might be because the LHA subscale Self-directed aggression was significantly related to DSH at *p* = .000 and the other subscales, aggression and antisocial behavior (*p* = .235 and *p* = .403, respectively), were not.

#### Predictors of DSH

Simple (Table 2) and multiple (Table 3) logistic regressions were performed with one controlling for age at participation. Calculating sensitivity and specificity, childhood ADHD correctly classified 76.4% of all participants with a sensitivity of 0% and a specificity of 100%, resulting in a positive predictive value of 0%. The amount of variance explained by the model was 2%. For early life adversities, the model correctly classified 76.6% of all participants with a sensitivity of 0% and a specificity of 100%, resulting in a positive predictive value of 0%. The amount of variance explained by the model was 4–6%. In the multiple model, only anxiety disorders, mood disorders, and having been bullied at school remained significant predictors of DSH.

**Table 2:** Predictors of deliberate self-harm behavior (simple logistic regression).

						95.0% C.I. for Odds Ratio	95.0% C.I. for Odds Ratio
Predictor	B	S.E.	Wald	*p*	Odds Ratio	Lower	Upper
**Alcohol SUD**	.84	.30	7.90	.005	2.3	1.92	4.19
**Cannabis SUD**	.03	.34	.01	.911	1.04	.05	2.05
**Sedative SUD**	.61	.29	4.21	.040	1.84	1.02	3.30
**Amphetamine SUD**	.81	.30	7.21	.007	2.25	1.24	4.06
**Hallucinogen SUD**	.67	.29	5.06	.024	1.96	1.09	3.52
**Metadon SUD**	.91	.37	6.11	.013	2.49	1.20	5.15
**Neurodevelopmental disorders**	.60	.33	3.21	.073	1.83	.94	3.55
**Borderline intellectual functioning**	.58	.34	2.89	.089	1.79	.91	3.51
**ADHD childhood any**	.62	.32	3.67	.055	1.86	.98	3.51
**ADHD adulthood any**	.82	.29	7.77	.005	2.28	1.27	4.08
**Anxiety disorders**	1.80	.36	24.77	.000	6.06	2.98	12.32
**Mood disorders**	2.28	.42	28.94	.000	9.86	4.28	22.71
**Childhood exposure to violence at home, repeated occ.**	1.06	.31	11.48	.001	2.89	1.56	5.34
**Violent offenses total**	-.57	.35	2.59	.107	.56	.28	1.13
**Bullied during school years**	1.36	.31	18.95	.000	3.91	2.11	7.22
**Number of psychosocial adversities**	.38	.12	9.72	.002	1.47	1.15	1.87
**Violence against parents repeated occ.**	.84	.34	6.13	.013	2.32	1.19	1.89

**Table 3:** Predictors of deliberate self-harm behavior (multivariate logistic regression).

**Predictor**	Odds Ratio (95% C.I)	*p*	Adjusted Odds Ratio (95% C.I) ^a^	*p*
**SUD**	1.096 (.39–3.04)	.860	1.087 (.39–3.04)	.873
**ADHD adulthood, any**	1.327 (.67–1.63)	.418	1.413 (.70–2.83)	.331
**Anxiety disorders**	3.087 (1.38–6.92)	.006	2.811 (1.23–6.40)	.014
**Mood disorders**	4.950 (2.02–12.14)	.000	4.991 (2.01–12.35)	.001
**Bullied during school years**	3.123 (1.53–6.36)	.002	3.270 (1.59–6.71)	.001
**Childhood exposure to violence at home, repeated occ.**	1.370 (.67–2.79)	.386	1.280 (.62–2.63)	.502

^a^ Adjusted for age

## Discussion

This study reports findings on associations of DSH with clinical, neurocognitive, psychosocial, and criminological factors in a group of male violent offenders in Sweden, 18–25 years old. The prevalence of DSH in this study was 23% (n = 62), with a mean age of onset in the late adolescent years. DSH was mainly associated with mood disorders, anxiety disorders, AD symptoms, repeated exposure to violence during childhood, and having been bullied during childhood. Among the 62 self-harming offenders, many reported a repetitive DSH behavior and only 6 offenders admitted their DSH act had a suicidal intent. This study provides evidence of an increased prevalence of DSH in people exhibiting interpersonal aggressive behaviors and of DSH as an indicator of psychological ill-health not only in women, but also in men.

### Prevalence and character of DSH among young violent offenders

Previous studies on non-clinical populations with a comparable sample size have estimated the prevalence of DSH at 4% to 13% [5, 45]. Studies showing a higher prevalence have used the NSSI definition, which does not include suicide attempts. In this study, an increased prevalence of DSH was found with a total of 62 (23%) deliberately self-harming offenders. The majority of offenders reported a repetitive DSH behavior, which is not consistent with previous studies in the general population where only a minority (10%) repeated DSH behavior [4]. This finding is not entirely unanticipated since this group distinguishes itself from the general population in several ways e.g., growing up under severe circumstances [33], something that previously has been linked to increased risk of DSH [25, 26, 27]. The mean age of onset is comparable to that in the general population, although in some cases in our study DSH was reported in the early childhood years, further indicating unstable circumstances and mental ill-health during childhood.

### Characteristics associated with DSH among young violent offenders

#### Clinical

There was a significant positive association between mood and anxiety disorders and DSH in accord with previous research [46]. In the group of DSH participants (n = 62), 82% (n = 50) suffered from some kind of anxiety disorder and 89% (n = 55) from a mood disorder. Self-harm has previously been described as an emotion regulator and as an act of tension release that, for a limited time, eases an individual’s temporary distress [47]. A study of a comparable group of 173 male prisoners concluded that 31.6% of the prisoners used DSH to obtain anxiety release and 21% to release anger [27], which both fall under Gratz’s definition of emotion regulation [47]. In the present study, mood disorders and anxiety disorders predicted the risk of engaging in DSH by 9.9 and 6.1 times, respectively, with statistical significance. Childhood ADHD was not a significant predictor of DSH, and did not result in a sensitive prediction model in this group of offenders. Taking into account possible confounders in a multiple logistic regression (Table 3), the increases in risk remained significant, but decreased to 3.1 and 4.9 times, respectively. Controlling for age at participation only resulted in minor differences, not considered as clinically relevant. Since motives for DSH were not investigated beyond whether or not there was suicidal intent, the study offers no conclusions on the functions of the behavior. DSH is often related to borderline personality disorder [48]; however, due to the low prevalence of borderline personality disorder among the participants in this study, this association could not be tested. However, DSH was found to be related to symptoms of both AD and HD, in line with previous research [29], indicating a relationship with disinhibited behavior and attention deficits. However, since very high rates of mood disorders and anxiety disorders were noted among the DSH offenders, the question is whether the observed attention difficulties might be due, at least in part to comorbid depression or anxiety. Contrary to previous findings, conduct disorder was not related to DSH in the present study. This finding could be due to the high prevalence of conduct disorder in the sample, providing too little variation in the analyses.

Alcohol use disorder was significantly more prevalent among DSH offenders, in line with previous research findings showing alcohol use disorder as a risk factor for suicide [49]. Other authors have discussed the combination of depression and alcohol and its prediction of suicide [50]. From results of the present study, we argue that DSH is associated to alcohol abuse; however, it is not possible to distinguish whether alcohol use disorder is causally related to DSH or if it acts as a moderator. However, other studies have linked DSH acts directly to episodes of alcohol use [51]. In the simple logistic regressions, all SUD except cannabis could significantly predict DSH with odds ratios from 2.0 to 2.5. The risk of engaging in DSH was twice as high for offenders with any SUD. In contrast to other studies [52, 53] DSH could not be predicted by cannabis SUD. This might be due to lack of variation in this sample as the majority (77%) of the offenders had cannabis SUD. Whereas previous studies have established the association between cannabis and DSH, the results of the present study show that this association may not be causal, and that instead, one should consider what other underlying risk factors cannabis and DSH share.

#### Cognitive functioning

In this study, DSH was not related to general intellectual ability. However, DSH participants scored significantly lower on the POI scale than offenders without DSH, indicating possible deficiencies in visual tracking, perceptual organization, and reasoning among offenders with DSH. These findings could possibly be associated with problems of visual attention, as indicated by the increased prevalence of AD symptoms among the DSH participants. Whether this is due to actual visuo-perceptive dysfunction or comorbid mental illnesses affecting attention (e.g., anxiety and depression), remains to be investigated in future research.

#### Psychosocial

Research has shown that participants who have experienced abuse during childhood are more likely to report suicide attempts [54]. However, in other studies, individuals who have attempted suicide have shown significantly higher rates of impulsivity and aggression, and the question remains whether being abused during childhood or underlying traits of aggression and impulsivity construct a risk of DSH [55]. Impulsivity is one of the traits in the HD symptoms which were also significantly associated to DSH in this study. This shows how complex the relationships between factors are, and causal associations are almost impossible to detect at group level. In the present study, 52% of the offenders had been exposed to violence during childhood [33]. Violence in the early years is considered a trauma [25], which is a strong predictor of DSH [26]. In this study, repeated exposure to violence during childhood was significantly more prevalent in the DSH group. When the variable exposure to violence at home was analyzed singularly in a simple logistic regression, the prediction was highly significant (*p* = .001), but when the other factors were taken into account (see Table 3), exposure to violence was no longer a significant predictor. However, the dose-response relationship between early adverse experiences and DSH in the results indicates that although other variables were more important predictors of DSH in this sample, increased number of psychosocial adversities during childhood were linked to adverse outcomes in adulthood, including DSH. Yet, in this group of offenders, only using early life adversities as a predictor did not result in a sensitive model why one should consider more than only this factor when predicting DSH.

### Limitations

It is unwise to draw conclusions about the role of DSH in offenders’ mental health from this study since there is little variation in the sample, which is not representative of the general population either. Thus, generalizations should be made with care and preferably only about young male violent offenders. Also, the low prevalence of some disorders commonly associated with DSH made it impossible to test their associations with DSH in this sample. Finally, the effect sizes of associations detected were small to medium, indicating that the tested variables are only part of the puzzle of why some people direct their aggressive behaviors toward themselves as well as others.

### Clinical implications

This study shows that violent offenders are more likely than the general population to direct their aggressive behaviors not only toward other people, but also toward themselves. Many of those offenders who harm themselves do so on multiple occasions, although often without a firm intention of committing suicide. However, it is clear from their suicidal acts that whether or not they truly wish to kill themselves, many of the offenders displaying DSH are at very high risk of dying by suicide at some point. The age of onset of DSH in the current study group, in combination with how many of them were placed in institutions during childhood or adolescence, indicates that DSH should be constantly assessed and managed in youth correctional institutions and prison and probation services. Even if DSH does not predict completed suicide in all DSH individuals, it must be taken seriously as an indicator of mental health problems among young violent offenders.

Possible models to prevent DSH among offenders, aside from treatment of mood disorders and anxiety disorders [45], could be substance use prevention programs. For the majority with SUD, this might have a beneficial effect on several risk factors simultaneously as such programs have been found to ameliorate not only SUD, but also mood and anxiety disorders [56]. Depending on the function of the self-harming behavior among offenders, which remains to be investigated, psychological treatments such as the Dialectical Behavior Therapy forensic model [57] might be suitable for those with emotion regulation deficiencies as it is essential that these individuals are taught strategies to regulate their emotions and communicate their feelings without harming their bodies. Emotional dysregulation is one theoretical common denominator for both DSH and overt aggression, where over-regulation or under-regulation of emotions might increase the risk of dysfunctional behavior such as DSH or aggression [58, 59]. A study of the function of DSH found that DSH is strongly associated with a shift in emotions, where the participants in the study felt overwhelmed, sad and frustrated before the self-harming act and relieved and calm after. [60] That is, the DSH acted as a coping strategy, providing relief from negative emotions [61, 62]. In a recent study, emotional dysregulation and trait impulsivity predicted both aggression and DSH [59], and there is also evidence for an interaction between emotional dysregulation, trait anger, and aggression [63]. Considering the participants in the current study, it is quite possible that emotional dysregulation might influence the occurrence of both DSH and aggression, even though this could not be investigated in the current study. With regard to their adverse psychosocial backgrounds and the fact that they are imprisoned at the time of the study, it is also plausible that the offenders had had only a few, if any, opportunities to develop more functional emotion regulation skills. Yet, the interactions between emotion regulation, DSH, and aggressive behavior remains to be investigated, and further knowledge on the function of both DSH and aggression in forensic populations is urgently needed in order to provide suitable preventive interventions against these harmful behaviors.
